# New Article for Pessary

**Published:** 1848-06

**Authors:** 


*New Article for Pessary.*—We take the following extract from a review in the Western Lancet:—

"Many articles may be used as pessaries besides those named by the author. One of the latest we have seen, is an egg-shell—prepared by making a small hole in each extremity of the egg, and blowing out its contents. It is light and smooth, and when well placed with the point looking towards the pubis, answers all the purposes of an "instrument of two inches." Care must be taken to use the shell, not the egg. Inattention to this point has given rise to painful and living results. In one case an egg was introduced into the vagina, and at the end of the usual period of incubation, the patient felt such pointed suffering, that she was induced to remove her "uterine splint," when lo! and behold! there was the bill of a living chick. The practitioner had overlooked the fact that some places, are the best places in the world for hatching."

